# Piloting the Global Subsidy: The Impact of Subsidized Artemisinin-Based Combination Therapies Distributed through Private Drug Shops in Rural Tanzania

**DOI:** 10.1371/journal.pone.0006857

**Published:** 2009-09-02

**Authors:** Oliver J. Sabot, Alex Mwita, Justin M. Cohen, Yahya Ipuge, Megumi Gordon, David Bishop, Moses Odhiambo, Lorrayne Ward, Catherine Goodman

**Affiliations:** 1 Malaria Control Team, Clinton Foundation HIV/AIDS Initiative, Boston, Massachusetts, United States of America; 2 National Malaria Control Program, Ministry of Health and Social Welfare, Dar es Salaam, Tanzania; 3 Center for Strategic HIV/AIDS Operations Research, Clinton Foundation HIV/AIDS Initiative, Boston, Massachusetts, United States of America; 4 Tanzania Country Office, Clinton Foundation HIV/AIDS Initiative, Dar es Salaam, Tanzania; 5 Mott MacDonald Group Limited, Croydon, United Kingdom; 6 Social Research Division, Steadman Group, Nairobi, Kenya; 7 Kennedy School of Government and Harvard Business School, Harvard University, Cambridge, Massachusetts, United States of America; 8 Health Policy Unit, London School of Hygiene and Tropical Medicine and Kenya Medical Research Institute/Wellcome Trust Research Programme, Nairobi, Kenya; BMSI-A*STAR, Singapore

## Abstract

**Background:**

WHO estimates that only 3% of fever patients use recommended artemisinin-based combination therapies (ACTs), partly reflecting their high prices in the retail sector from where many patients seek treatment. To overcome this challenge, a global ACT subsidy has been proposed. We tested this proposal through a pilot program in rural Tanzania.

**Methods/Principal Findings:**

Three districts were assigned to serve either as a control or to receive the subsidy plus a package of supporting interventions. From October 2007, ACTs were sold at a 90% subsidy through the normal private supply chain to intervention district drug shops. Data were collected at baseline and during intervention using interviews with drug shop customers, retail audits, mystery shoppers, and audits of public and NGO facilities.

The proportion of consumers in the intervention districts purchasing ACTs rose from 1% at baseline to 44.2% one year later (p<0.001), and was significantly higher among consumers purchasing for children under 5 than for adults (p = 0.005). No change in ACT usage was observed in the control district. Consumers paid a mean price of $0.58 for ACTs, which did not differ significantly from the price paid for sulphadoxine-pyrimethamine, the most common alternative. Drug shops in population centers were significantly more likely to stock ACTs than those in more remote areas (p<0.001).

**Conclusions:**

A subsidy introduced at the top of the private sector supply chain can significantly increase usage of ACTs and reduce their retail price to the level of common monotherapies. Additional interventions may be needed to ensure access to ACTs in remote areas and for poorer individuals who appear to seek treatment at drug shops less frequently.

**Trial Registration:**

Controlled-Trials.com ISRCTN39125414.

## Introduction

Artemisinin-based combination therapies (ACTs) have become a mainstay of malaria treatment because of their high efficacy and their potential to delay the development of antimalarial resistance [1], [2]. Yet despite availability of substantial donor funding, the proportion of fevers treated with ACTs remains minimal [3], [4]. This partly reflects limited ACT availability outside the public sector. In many countries, 40–60% of people seek treatment for fever or malaria from private vendors, such as pharmacies, drug shops and general stores [5]. However, ACTs are typically sold at retail prices 20–40 times those of common alternatives such as amodiaquine and sulphadoxine-pyrimethamine (SP), restricting their uptake by consumers, particularly in rural areas [3], [6], [7]. As a result, most retail sector anti-malarial customers continue to use older therapies for which widespread resistance has been reported or artemisinin monotherapies, which are strongly discouraged by the World Health Organization because their use is likely to accelerate the development of artemisinin resistance [8], [9]. Many others purchase antipyretics only.

Anticipating this challenge, in 2004 the Institute of Medicine recommended a global subsidy of ACTs as the best means to achieve high coverage and prolong the efficacy of these drugs [10]. It argued that reducing the ex-factory price of ACTs to that of common alternatives (roughly $0.05) would ensure their widespread distribution through private channels and crowd out other drugs. This concept was further developed by the Roll Back Malaria Partnership and launched by the Board of the Global Fund in November 2008 as the Affordable Medicines Facility-malaria (AMFm).

The AMFm is scheduled to be launched in 11 countries within the coming year. However, the dearth of evidence on the likely impact of such a subsidy has hindered its design and raised numerous concerns about investing public money in an unproven mechanism [11]. To fill this evidence gap, we piloted the AMFm model in two rural Tanzanian districts starting in October 2007. This report presents the results from the pilot and assesses their implications for implementation of the AMFm and other large-scale subsidies.

## Methods

### Study design

**Figure 1:**
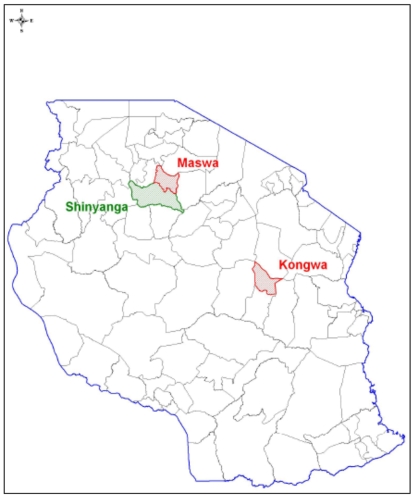
Location of project districts by role in study. The red areas denote the two intervention districts, while the green area shows the control district.

The intervention was conducted in two rural districts of Tanzania: Maswa in Shinyanga region and Kongwa in Dodoma region. A third district, Shinyanga Rural in Shinyanga region, served as a control (see Figure 1). We conducted a detailed analysis of all districts in the country according to a range of key indicators, including malaria endemicity, population per health facility, employment, prevalence of private drug shops, and bed net ownership. The selected districts were among the few roughly comparable across all indicators, with high malaria transmission, large numbers of private drug shops and, importantly, no malaria-related trials (e.g., vaccines) underway. Socioeconomic status in the three districts was below national averages as evidenced by comparison of key assets such as housing materials, toilet facilities, and availability of electricity [12]. The selected districts were randomly assigned to one of the three arms in the study design: subsidy, subsidy plus suggested retail price, and no subsidy (control). As two of the qualified districts were adjacent, randomization was limited so that one of the adjacent districts served as the control.

The project centered on the distribution of ACTs at highly subsidized prices. The project managers procured quantities of artemether-lumefantrine (AL), the recommended first-line ACT in Tanzania, from the manufacturer, Novartis, and sold them to a pharmaceutical wholesaler in Dar es Salaam at an average of $0.11 per dose, 88% below the price offered to public buyers (private buyers are typically offered substantially higher prices). The wholesaler, Nufaika Distributors Limited, was selected due to its extensive distribution network and interest in selling ACTs, among other factors, following due diligence of 10 similar businesses. The wholesaler received no instructions other than to sell the ACTs to drug shops in the two intervention districts according to its standard practices. It was made clear that the wholesaler would not be monitored or held accountable for its pricing, stocking, or other practices.

Small drug shops, known as *duka la dawa baridi* (DLDB), were the primary retail outlet for subsidized ACTs in the project. Past studies have found that these shops are the most important commercial source of anti-malarials in Tanzania and it is estimated that there are more than 8,000 in operation nationwide in rural and urban areas. Under Tanzanian law, these shops are permitted to sell over-the-counter medicines, but not products requiring a prescription (an exception was granted for ACTs for this project) [13], [14]. Small volumes of anti-malarials are also sold through general stores alongside common staples, but distribution to these outlets was not encouraged as they are not legally permitted to sell pharmaceuticals.

AL was distributed in four weight-specific packs, in redesigned packaging with simplified dosing instructions in the local language, Kiswahili. ACTs distributed to Kongwa were marked with a suggested retail price (SRP) intended to inform consumers of the maximum amount they should pay. The SRP was set at 300, 600, 900, and 1200 Tanzanian Shillings (0.25, 0.50. 0.75. and 1 USD respectively) for the four weight packs respectively based on an analysis of costs in the supply chain; no SRP was included on drugs distributed to Maswa in order to test its effect on price outcomes.

The AMFm will support countries to conduct accompanying interventions such as training, behavior change communication (BCC), and regulatory strengthening to facilitate the effective distribution and use of subsidized ACTs [15]. This project accordingly included some of those activities in the intervention districts. Prior to the initiation of the subsidy, the Tanzania Food and Drug Authority (TFDA) conducted a one-day training of DLDB attendants focusing on malaria symptoms and ACT dispensing and dosing. Population Services International conducted a range of BCC activities, including local radio advertisements, wall paintings, and themed cultural shows, throughout the project. The activities emphasized the importance of using ACTs and their availability in private shops, as well as basic messages on the dangers of malaria and the importance of prompt treatment-seeking.

### Data collection

The study's primary outcomes were the proportion of antimalarial consumers visiting DLDB who purchased subsidized ACTs and the price they paid for the drugs. Secondary outcomes included the proportion of DLDB stocking the product, the socioeconomic status of the consumer, the age of the intended patient, and ACT distribution by public facilities during the same period.

We employed four data collection methodologies: exit interviews, retail audits, mystery shoppers, and public facility audits. All four methodologies were conducted together five times during the project: once prior to the launch of the subsidy in August 2007 and four times throughout the intervention in November 2007 and March, August, and November 2008. Data were collected at all DLDB and public facilities in the three districts. DLDB were initially identified through TFDA records, with unregistered shops captured through discussions with local informants and systematic physical reconnaissance throughout each district. Geographical positions of all shops were recorded using hand-held GPS units (Garmin Etrex).

For the exit interviews, data collectors positioned themselves near a DLDB and remained there for the full business day. All customers emerging were asked to answer a short questionnaire on the products bought, and the brand of the product was visually verified. To assess the customers' socio-economic status, interviewees were asked a series of questions about their household assets from the 2003–04 Tanzania HIV/AIDS Indicator Survey.

DLDB retail audits were conducted twice during every data collection period, at a four week interval. Collectors recorded the volume of all anti-malarial stocks present. A short questionnaire was also administered to the owner or attendant to determine the amount of each product newly purchased and disposed of (e.g., due to expiry or damage) during the previous four weeks. Stock levels were then compared between the two audits and purchases and disposals subtracted to determine the volume of sales during that period.

DLDB pricing and dispensing practices were also observed by having collectors visit each shop once per survey posing as a consumer seeking malaria treatment. Two such “mystery shopper” scenarios were employed – adults purchasing for themselves and purchasing for a nine-month old child at home with malaria symptoms. Data collectors also visited all public and non-governmental organization (NGO) health facilities in each survey period to review ACT stocks and dispensing records.

In this paper we focus primarily on data from the baseline in August 2007 and follow-up in August 2008 because of potential seasonal variation in malaria transmission and treatment seeking. To enable robust analysis of the impact of the SRP, pricing data are pooled across intervention surveys and compared to the baseline. A full set of results for all data collection periods is available at http://www.cshor.org/TanzaniaPilotData.xls.

### Data analysis

To assess geographical variation in outcomes, the competition level of all DLDB was calculated using the fixed radius approach [16]. The competitive space of each DLDB was defined as 1 kilometer and each shop was assigned to a competition index category between 0 and 5 based on the number of other DLDB within that radius.

Exit interviewees were allocated to wealth quintiles using the asset weights and quintile break-points generated through principal components analysis of 53 variables from the 2003–2004 HIV/AIDS Indicator Survey [17]. Due to operational challenges associated with administering asset ownership questions at shops (as opposed to at household level as is typical), several variables from the original survey were not captured, including additional forms of household lighting, water source, and land ownership status. Asset weights and national quintiles were therefore calculated using only those variables captured in the exit interviews.

To enable comparison of price across products, the exact number of pills purchased (syrups and injectables were excluded) by interviewees or mystery shoppers was recorded and the price paid for a full appropriate dose for the intended patient then extrapolated using the standard dosing schedule according to Tanzania national treatment guidelines or product registration with the TFDA [18]. Similarly, to compare sales across products, we converted the total number of pills sold to adult equivalent doses based on the recommended dosing.

Survey data were analyzed using SPSS v.14/16 (Chicago, Illinois) and SAS v.9.1 (Cary, NC), and GPS data using MapInfo v.7.8 (Troy, New York). Proportions were compared using chi-square tests. Student t-tests were used to compare means and the Satterthwaite t-test was used when variances were significantly unequal. A repeated measures multivariate regression model was used to compare differences in purchase price while controlling for potentially confounding factors and adjusting for clustering of multiple purchases in the same shops.

### Ethical Considerations

As a pilot project of the Tanzania Ministry of Health and Social Welfare to prepare for a national program, the interventions were developed with the Tanzania Food and Drug Authority and National Malaria Control Program according to the policies and guidelines of the Ministry and approved by the Chief Medical Officer accordingly. No additional interventions were added as part of the subsequent evaluation. Oral informed consent was obtained from all consumers emerging from drug shops as well as from drug shop owners prior to the administration of retail audits. No ethnic or individual identifying information was captured. This study complied with the guidelines of the Declaration of Helsinki.

## Results

Table 1 summarizes the observations recorded for each methodology. The total number of DLDB audited increased from 200 in August 2007 to 216 in August 2008 due to the opening of new shops, with 30 (13%) and 39 (15%) additional shops closed or refusing to participate at these two time periods respectively. A similar number of all shops observed were in areas of high competition (36% and 38% in categories 4 and 5 in August 2007 and August 2008 respectively) as in areas of low competition (35% in categories 0 and 1 in both periods). High competition DLDB were located in population centers while those in low competition categories were 20–25 km from major roads. The majority of consumers interviewed in all districts in August 2007 and August 2008 were from the two least poor SES quintiles (59% and 68% respectively).

**Table 1:** Recorded observations by methodology, characteristic, and district, August 2007 and August 2008.

	August 2007			August 2008		
	Maswa	Kongwa	Control	Maswa	Kongwa	Control
**DLDB Audited**	**73**	**60**	**67**	**83**	**68**	**65**
Comp Index 0	12	7	15	13	20	14
Comp Index 1	12	15	9	10	7	12
Comp Index 2–3	21	18	20	28	18	13
Comp Index 4–5	3	5	6	0	10	18
Comp Index 5+	25	15	17	32	13	8
**Exit Interviewees**	**346**	**53**	**181**	**167**	**288**	**118**
Buying for Ages 16+	275	37	125	79	212	76
Buying for Ages 5–16	28	7	10	39	42	10
Buying for Ages<5	43	9	46	49	34	32
SES Quintile 1 (poorest)	3	4	21	8	12	4
SES Quintile 2	27	7	40	16	9	8
SES Quintile 3	62	8	66	32	50	9
SES Quintile 4	137	26	45	56	112	52
SES Quintile 5 (least poor)	117	8	9	55	105	45
**Mystery Shoppers**	73	60	67	81	65	65
**Public/NGO facilities audited**	38	33	34	35	36	36

### Stocking

There was a pronounced increase in the proportion of shops stocking ACTs in the intervention districts, from 0/133 in August 2007 to 109/151 (72.2%) in August 2008 (p<0.001). Shops stocking ACT in the control district changed negligibly from 1/67 (1%) to 0/65 over the same period. Shops with two or more other shops in their competition radius were significantly more likely to stock ACTs in August 2008 (82/101, 81.2%) than those with 0 or 1 competitor (27/50, 54.0%; p<0.001). By comparison, stocking of some other common anti-malarials was more consistent across competition categories: 75/101 (74.3%) of shops in category 2 and above and 34/50 (68.0%) in categories 0 and 1 stocked amodiaquine, a non-significant difference.

### Pricing

**Figure 2:**
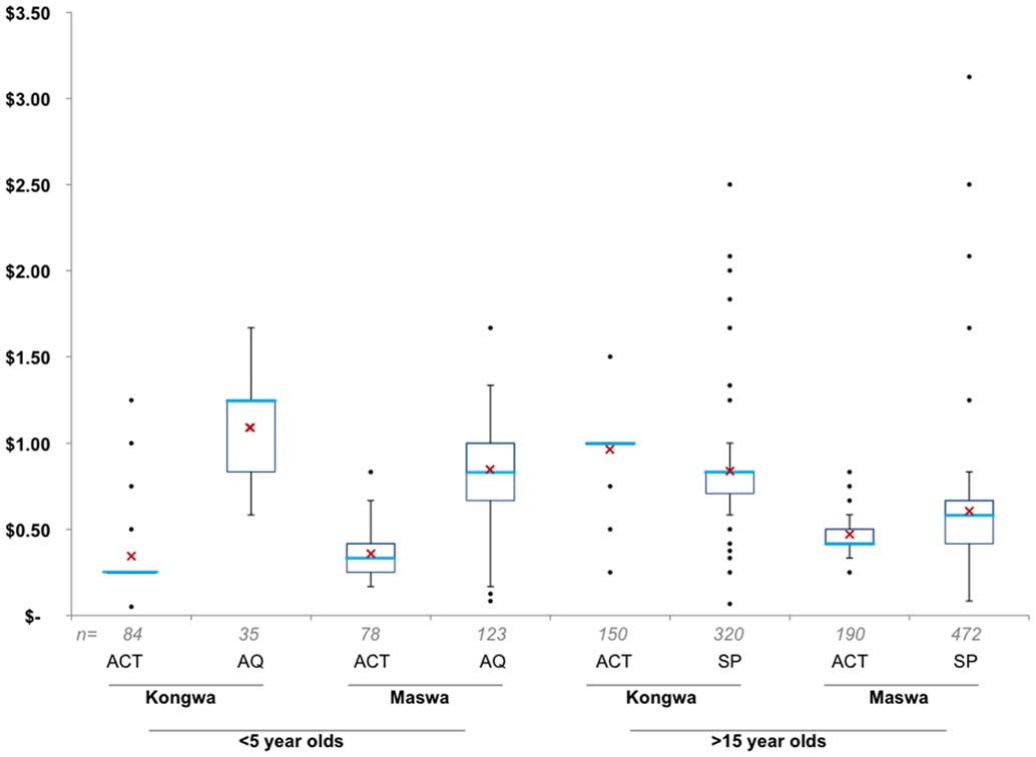
Price paid for ACTs and most common alternative anti-malarials by interviewed consumers. Prices of subsidized ACTs and the most commonly purchased alternative anti-malarial – amodiaquine (AQ) or sulphadoxine-pyrimethamine (SP) – observed in the two intervention districts between November 2007 and November 2008 are displayed by intervention district and age of intended recipient. The thick blue line denotes the median and the red X the mean, with the surrounding box delineating the interquartile range (IQR). The lines extending from each box mark 1.5 times the IQR, with dots showing outliers that do not fall within this range.

Interviewed consumers paid a mean price of $0.58 for all ACTs (SD = $0.28) during the study period (Figure 2). Consumers purchasing ACTs for children under 5 paid significantly less than those buying for adults (16+ years), at a mean expenditure of $0.35 (SD = $0.19) and $0.70 (SD = $0.28) respectively (p<0.001). Overall, the average price paid for ACTs for adults did not differ significantly from expenditures on SP ($0.67, SD = $0.34), but was significantly higher than for amodiaquine ($0.48, SD = $0.27; p<0.001). The mean price paid for ACTs for children under 5 was significantly less than for both SP ($0.51, SD = $0.28; p = 0.001) and AQ ($0.86, SD = $0.30; p<0.001). Controlling for the age of the intended recipient, the district in which the drug was purchased, and clustering of multiple purchases in shops, the price paid for ACTs did not vary significantly by either the SES quintile of the consumer or the competition category of the shop.

Consumers paid significantly more for the three largest of the four AL weight packs in Kongwa than in Maswa (all p<0.001), while no significant difference was observed for the lowest (5–15 kg) dose. In Kongwa, the mean price paid for two AL doses, infant (5–15 kg) at $0.34 (SD = $0.24; p<0.001) and child (15–25 kg) at $0.55 (SD = $0.15; p = 0.034), were significantly higher than the SRP. Mean prices did not differ significantly from the SRP for the other two doses.

### Uptake and Sales

Table 2 presents exit interview data on uptake for AL, artemisinin monotherapy, and the two other most commonly purchased antimalarials, amodiaquine and SP. The proportion of anti-malarial consumers in the intervention districts who purchased ACTs increased strikingly during the project, from 4/399 (1.0%) in August 2007 to 201/455 (44.2%) in August 2008 (p<0.001). This proportion subsequently declined insignificantly to 227/572 (39.7%) in November 2008. Over the same period, purchases of SP and amodiaquine in the intervention districts declined significantly, from 232/399 (58.2%) to 163/455 (35.8%; p<0.001) and 146/399 (36.6%) to 75/455 (16.5%; p<0.001) respectively. Use of artemisinin monotherapies remained negligible throughout the study.

**Table 2:** Purchase of anti-malarials by exit interview customers by district, August 2007 and August 2008.

	August 2007				August 2008			
	Maswa	Kongwa	Total Intervention Districts	ControlDistrict	Maswa	Kongwa	Total Intervention Districts	Control District
**Adults (ages 16+)**								
*Antimalarial purchases of which:*	275	37	312	125	79	212	291	76
ACT	3 (1%)	1 (3%)	4 (1%)	0 (0%)	38 (48%)	66 (31%)	104 (35%)	0 (0%)
SP	187 (68%)	26 (70%)	213 (68%)	78 (62%)	30 (38%)	118 (56%)	148 (51%)	63 (83%)
AQ	71 (26%)	10 (27%)	81 (26%)	41 (33%)	8 (10%)	23 (11%)	31 (11%)	12 (16%)
**Children (ages<5)**								
*Antimalarial purchases of which:*	37	6	43	35	49	34	83	32
ACT	0 (0%)	0 (0%)	0 (0%)	0 (0%)	20 (41%)	24 (71%)	44 (53%)	2 (6%)
SP	1 (3%)	2 (33%)	3 (7%)	3 (9%)	1 (2%)	2 (6%)	3 (4%)	3 (9%)
AQ	35 (95%)	4 (67%)	39 (91%)	32 (91%)	22 (45%)	8 (24%)	30 (36%)	22 (69%)

Purchases by mystery shoppers followed a similar trend, with the proportion of shoppers offered ACTs in intervention districts rising from 6/133 (4.5%) to 83/146 (56.9%; p<0.001) and those offered SP declining from 83/133 (62.4%) to 38/146 (26.0%; p<0.001). When restricted to only shops stocking ACTs, the proportion of interviewed consumers and mystery shoppers buying ACTs was significantly higher than when all shops were included: 55.5% v. 44.2% (p = 0.001) and from 76.4% v. 56.9% (p = 0.001) respectively. No change in ACT purchasing was observed in the control district.

In August 2008, 44/83 (53.0%) of consumers purchasing anti-malarials for a child under 5 bought ACTs compared to 104/291 (35.7%) of those purchasing for an adult (p = 0.005). There was no correlation between the SES of the consumer and the likelihood of buying ACTs, with ACTs comprising 44.4% of purchases by consumers in the poorest two quintiles (n = 45) compared to 42.4% by those in the least poor quintiles (n = 328). Similarly, there was no significant difference in the proportion of consumers buying ACTs between high and low competition stores.

**Figure 3:**
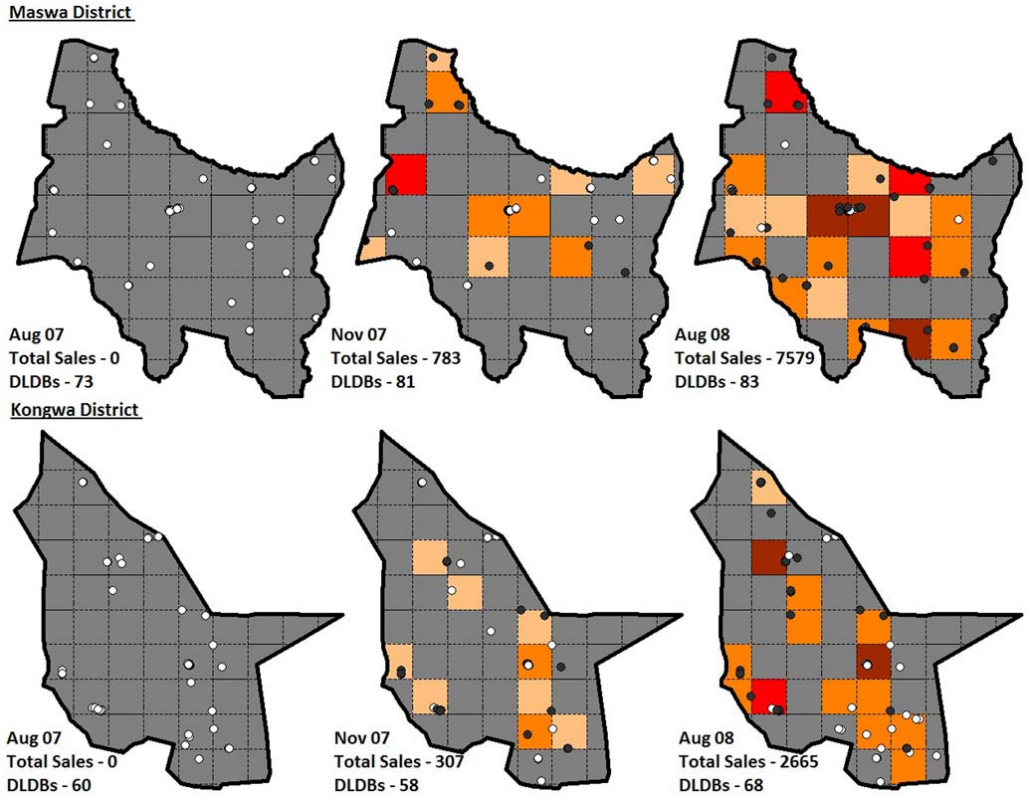
Stocking and sales of subsidized ACTs at drug shops in intervention districts. All *duka la dawa baridi* (DLDB) in Maswa (top) and Kongwa (bottom) districts are mapped as either white (ACTs not in stock at time of survey) or black (ACTs in stock) circles. Data is shown at baseline (August '07) and two periods during implementation (November '07 and August '08). Districts are divided into 10 km^2^ squares, with the total volume of adult equivalent doses of ACTs sold in that area over the preceding four weeks shown by the color of shading as follows: Tan = 1 to 50 doses sold; Orange = 51 to 250 doses; Light red = 251 to 500 doses; and Dark red = 501 to 10,000 doses.

Retail audits showed that 9,786 adult equivalent doses of ACTs (60.3% of all adult equivalent anti-malarial doses) were sold by DLDB in a 4-week period in July/August 2008, while ACT sales in the control district remained negligible (see Figure 3). Overall distribution of ACTs in the intervention districts, including dispensing from public and NGO facilities, increased 62% between November 2007 (the first collection following intervention) and August 2008 from 30,946 to 69,068 doses, with ACTs distributed through the private sector accounting for 38% of that growth.

## Discussion

The introduction of subsidized ACTs resulted in a rapid and pronounced increase in the proportion of people accessing ACTs from private shops from close to zero to 44% after one year. Distribution of ACTs through the public sector rose at the same time, indicating that the intervention contributed to increases in overall volumes of the drug distributed. Importantly, the greatest increases in ACT usage at DLDB were by those seeking treatment for children under 5, the group at the greatest risk of malaria mortality. However, as other studies have found, purchases at DLDB for children under 5 were modestly underrepresented compared to the estimated fever incidence for this age group [19].

The rise in ACT usage appears to have crowded out the use of some sub-optimal therapies such as SP and AQ, although a substantial number of consumers continued to purchase these drugs at the end of the study. There was no significant change in ACT uptake during the last 3 months of the study, but this does not suggest that a long-term plateau in ACT purchasing had been reached. The intervention is targeting a fundamental shift in the market for anti-malarials, which will require years to fully realize. As shown in other studies, poorer individuals appear to have sought treatment for malaria at DLDB substantially less frequently than wealthier ones, suggesting that additional interventions may be needed to increase ACT access among this population [20].

The subsidy had the intended effect of reducing the retail price of ACTs to levels similar to commonly used alternatives. On average, consumers paid 93% less than ACT prices regularly observed in private outlets in rural and urban areas across Tanzania [21]. Contrary to competition theory and common concern about the AMFm, consumers did not pay significantly more for ACTs at more remote shops facing less competition [16]. These results also contradict another common critique of large-scale ACT subsidies: that businesses would apply excessive mark-ups thereby minimizing the benefit to consumers. There were almost no instances of such “price gouging,” with 86% of all purchases within $0.08 of the SRP for the dose and the highest price paid still 81% below typical ACT prices in Tanzanian shops.

The SRP appears to have had the opposite of the intended effect, artificially inflating prices in Kongwa above those determined by the market in Maswa. The SRP levels were determined based on estimated costs and profit margins in the private anti-malarial supply chain derived from interviews with more than 50 businesses. That those levels were substantially above the retail market prices suggests that an SRP should be used with caution, based on a more detailed understanding of pricing practices and only in cases where unreasonable profit margins are being charged.

Although stocking of ACTs rose substantially during the intervention, it was skewed towards shops in towns and other population centers. When analysis was restricted to those shops stocking ACTs, purchases of the drug increased markedly, indicating that availability may have served as a major limitation on overall uptake. Many shopkeepers described high consumer demand for ACTs but expressed frustration at problems in obtaining the drugs. This suggests that addressing supply chain issues should be a central focus of large-scale subsidy plans. In particular, since wholesalers often lack an inherent financial incentive to distribute to remote outlets, public sector means of creating such incentives, such as providing a substantial rebate to wholesalers for achieving certain coverage levels in remote areas, should be explored [22].

Caution should be used in directly applying these findings to other settings. Socioeconomic factors, malaria treatment-seeking behavior, and the structure of private supply chains all vary widely between and within countries [5]. This study operated through only one wholesaler and one type of retail outlet, while national-scale subsidies will employ multiple of both. And although concerted measures were put in place to limit the Hawthorne Effect, it is possible that businesses and consumers were influenced by the presence of the study team.

Nevertheless, these results are cause for cautious optimism that subsidies applied at the top of the private supply chain can lead to rapid and dramatic increases in ACT usage. Consumer demand for ACTs was high and average retail prices remained low due to businesses applying modest mark-ups on the product, similar to those for older drugs such as SP and amodiaquine. The study also highlights key areas for further research on this topic. Overall changes in ACT coverage and treatment-seeking behavior, including among different SES groups, should be robustly assessed through studies collecting household level data. Moreover, the use of privately distributed ACTs for non-malarious fevers and opportunities for effectively introducing diagnosis into the private sector should be explored.

However, the need for further research should not delay implementation. No amount of piloting will fully recreate the conditions of a national or global subsidy, and some “learning by doing” will be inevitable. Our findings should provide sufficient confidence for large-scale implementation of the subsidy model as a central part of the global effort to increase ACT access from current dismal levels towards the Roll Back Malaria target of 80% of patients by the end of 2010. [6]
